# Isolation, Identification, and Antimicrobial Evaluation of Secondary Metabolite from *Serratia marcescens* via an *In Vivo* Epicutaneous Infection Model

**DOI:** 10.1021/acsomega.3c09522

**Published:** 2024-02-07

**Authors:** Çinel Köksal Karayildirim, Aslı Şahiner, Sennur Çalişkan, Fahri Emrah Soylu, Aylin Gökhan, Ebru Eroğlu, Yiğit Uyanikgil, Tamer Karayildirim

**Affiliations:** † Department of Biology, Science Faculty, Ege University, İzmir 35100, Turkey; ‡ Laboratory Animals Research Center, Ege University, İzmir 35100, Turkey; § Department of Histology and Embryology, School of Medicine, Ege University, Izmir 35040, Turkey; ∥ Department of Chemistry, Science Faculty, Ege University, Izmir 35100, Turkey

## Abstract

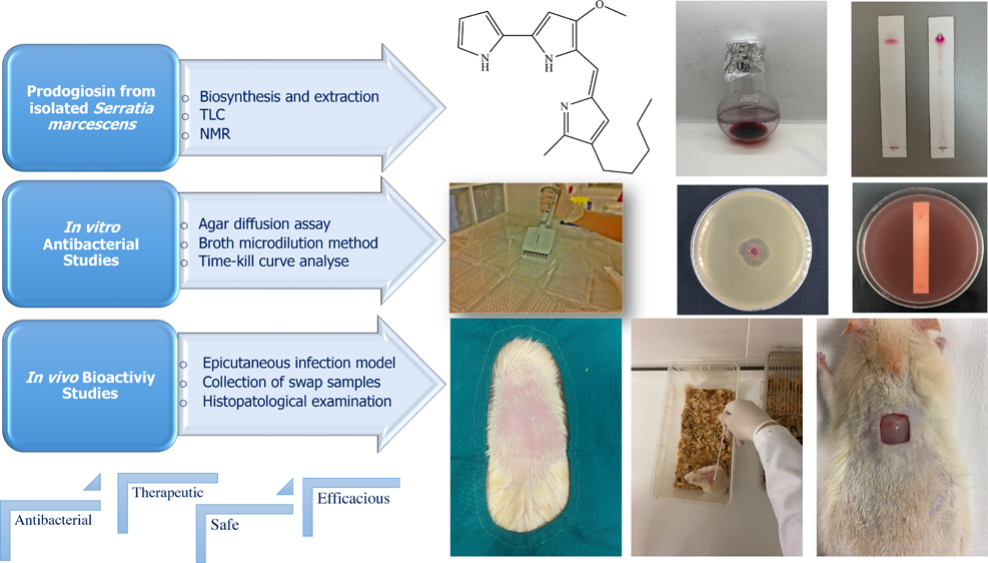

Microbial secondary metabolites, which play a pivotal role in struggling with infectious diseases, are the new source for controlling bacterial contaminations and possess a strong antimicrobial potential. The present study is designed to evaluate the *in vitro* and *in vivo* bactericidal activities of prodigiosin against *Staphylococcus aureus*. For this purpose, *Serratia marcescens* was used to produce prodigiosin. Characterization of the prodigiosin was carried out using NMR. In addition, bioautographic detection of prodigiosin was detected by TLC. Antibacterial assays, *in vivo* epicutaneous infection tests, swap analyses, and histopathological examinations were determined. The results revealed that prodigiosin was detected by NMR and TLC. According to antimicrobial susceptibility tests, prodigiosin is an efficient bactericidal compound that demonstrated strong antibacterial activity toward *S. aureus*. *In vivo*, animal studies determined that the strong inhibition of *S. aureus*-caused epidermal infection occurs by prodigiosin at 48 h. Histopathological results showed that *S. aureus* + prodigiosin skin sections consist of improved and healthy tissues without any infection area compared with the *S. aureus* and control groups. The *in vivo* study verified the antibacterial results with swap analyses, and histopathological findings showed that prodigiosin is a promising microbial metabolite effective against *S. aureus* infection. This study proved that prodigiosin with excellent bioactivity exhibited antibacterial properties, which might possess massive potential for new therapeutic approaches using micro-organisms.

## 1 Introduction

The Global Wound Care Market report declared that at the end of 2023, the global infection care, antimicrobial therapies, and the struggle with bacteria will have a value of approximately $26 billion.^1^ Bacterial skin and soft tissue infections are abundant worldwide, and many are caused by *Staphylococcus aureus*. It is well-known that *S. aureus* is a strong human pathogen that has adapted itself in response to selection pressure by the human immune system. *S. aureus* biofilm infections on open wounds have been widely characterized and depend on several factors, including the ability to adapt to environmental changes and produce a variety of molecules that contribute to virulence.

New therapeutic compounds and conventional drugs have entered the medical field in recent years with some adverse effects. These effects of existing drugs and multidrug resistance have become serious health issues that require the development of new antimicrobial agents.^2^ Various microbial strains have complex compounds with many biological activities. Because micro-organisms have developed therapeutic mechanisms to survive and struggle with stress conditions, they are a valuable source of bioactive natural products.

A micro-organism’s production of secondary metabolites is essential because it works as an inhibitor for others, or it can struggle with multidrug-resistant germs. The stress response of some micro-organisms in the form of the release of certain pigments has important properties exploited in the fields of biotechnology, mainly in agriculture, food, healthcare, and medicine. Secondary metabolites such as pigments have been shown to impart antitumor, antimicrobial, cytotoxic, and antihepatitis effects.^3^ Bacterial secondary metabolites such as prodigiosin gain more attention, and these pigments have many health benefits and biotechnological values.^4^

Prodigiosin is a red linear tripyrrole pigment and a member of the prodiginine family, which is usually produced and released by the *Serratia, Phaeocystis, Microcystis, Vibrio, Hahella,* and *Streptomyces* as a secondary metabolite.^5,6^ The tripyrrole red pigment has the ability to change color with changes in pH, allowing multifunctional applications.^7^ The literature has already described that prodigiosin is an active compound representing various protective and preventive activities.^3,8^ Although many *in vitro* studies evaluated prodigiosin efficacy, *in vivo* animal studies are very rare in the literature. For instance, it was demonstrated that prodigiosin was evaluated for the wound healing effect on *in vivo* rat models^9^ and it is affecting the nutrient metabolism of weaned rats^10^ Considering this, it is very important that the prodigiosin should be evaluated with *in vivo* animal studies for its antibacterial efficacy.

The main objective of this work is to prove the inhibition effect of prodigiosin, which is a secondary metabolite of *Serratia marcescens* against *S. aureus* infection. Thus, it was determined that microbial therapy has been proposed as an ideal strategy to reduce drug toxicity and improve treatment efficacy.

## 2 Materials and Methods

### 2.1 Materials

Tryptic soy agar (TSA), tryptic soy broth (TSB), and Mueller–Hinton agar were purchased from Sigma-Aldrich. Triphenyl-tetrazolium chloride (TTC) was supplied by Aldrich Chemical Co., U.K. Silica gel-coated aluminum TLC plates with flourescent indicator F254 were provided from Supelco. *S. marcescens* ATCC 14756, *S. aureus* ATCC 6538, and methicillin-resistant *S. aureus* (MRSA) ATCC 43300 were obtained from the Kwik-Stick (Microbiologics Inc., ABD). All the chemicals used for the experimental study are of highly analytical grade.

### 2.2 Methods

#### 2.2.1 Biosynthesis and Extraction of Prodigiosin from Isolated *S. marcescens*

The culture *S. marcescens* was cultivated in a 250 mL Erlenmeyer flask containing 50 mL of LB broth. The flask was inoculated with 10^6^ cfu/mL of *S. marcescens* and incubated for 72 h in a shaker (100 rpm) at room temperature. The culture was centrifuged at 10,000 rpm for 10 min, the supernatant was removed, and the pellet was used for further experiments. The cell pellet was suspended with acidified methanol (adjust to pH 3.0 using hydrochloric acid) and vortexed vigorously. Cell biomass was disrupted by ultrasonic treatment for 5 min. After methanolic cellular suspension, it was kept at +4 °C overnight followed by centrifugation at 10,000 rpm for 10 min to obtain a pigment-containing supernatant. The filtrate was concentrated by a rotary evaporator at 40 °C under vacuum.^11^

#### 2.2.2 Bioautographic Detection of Prodigiosin by Thin-Layer Chromatography

Thin-layer chromatography (TLC) bioautographic and agar diffusion methods were used to determine the prodigiosin as a target molecule.^12^ In order to purify prodigiosin, the crude extract was dissolved in methanol and subjected to silica gel column chromatography with the solvent system CHCl_3_/MeOH (9:1–5:5).^13^ The purity of the isolated compound was checked by TLC in a chloroform/methanol solvent system (1:1). According to the literature, the Rf value proved that prodigiosin was isolated and purified.^14^

TLC plate covers with Mueller Hinton agar (0.6%) containing 0.1% TTC and suspension at a final concentration of 10^7^ cfu/mL were developed. An inoculum of *S. aureus* in the 0.1% TTC containing a 2 mm layer of Muller–Hinton agar was distributed over the TLC plates. The plates were placed in a sterile tray, sealed to prevent the thin agar layer from moving, and incubated at 37 °C for 24 h. The bacterial inhibition zone was observed as clear areas against a pink-red-colored background.

#### 2.2.3 NMR Analysis

The structural elucidation of prodigiosin was performed by ^1^H NMR in CDCl_3_ on a Varian MERCURY plus-AS 400 NMR spectrometer (400 MHz) at room temperature.

#### 2.2.4 Antimicrobial Susceptibility Tests

##### 2.2.4.1 Bacterial Strain

Lyophilized Gram-positive *S. aureus* and MRSA cultures were selected for antibacterial assay. *S. aureus* was subcultured on TSB and incubated at 37 °C for 24 h prior to the antibacterial test. The bacterial suspension was adjusted to 0.5 MF using a densitometer (Grant Inst, Cambridge, U.K.). The antibacterial effects of the prodigiosin against *S. aureus* were analyzed using the agar diffusion method and time–kill assay.

##### 2.2.4.2 Agar Diffusion Assay

The agar diffusion method was employed to determine the antibacterial activity of prodigiosin against *S. aureus*. About 100 μL of culture suspensions was inoculated on Mueller–Hinton Agar media and spread using a sterile L-shaped glass rod. After drying the medium surfaces, 8 mm wells were punched using the tip of a sterile pipet. Then, 60 μL of prodigiosin was transferred to the wells and allowed to diffuse at room temperature for 2 h. Ampicillin (10 μg/mL) discs were used as the positive control, and DMSO-impregnated discs were used as the negative control. The inoculated plates were incubated at 37 °C for 24 h, and the diameters of the inhibition zones (mm) were measured.

##### 2.2.4.3 Broth Microdilution Method

The broth microdilution method was used to determine the minimum inhibitory concentration (MIC) values of prodigiosin according to CLSI M07-A9 (CLSI, 2021).^15^ The bactericidal activity test was evaluated using the Gram-positive test organism, which is *S. aureus*. To the first well, 80 μL of the prodigiosin was transferred and diluted by applying the 2-fold dilution rule. After that, 20 μL of micro-organism suspensions was inoculated into all wells at a final concentration of 10^6^. The medium and culture mixtures were used as a negative control. Ampicillin (10 μg/mL) solutions were used as positive controls. Microbial growth was visually determined after incubation for 24 h at 37 °C. To assess microbial viability following the incubation period, 2,3,5-triphenyl-tetrazolium chloride was introduced into each well. Red color formation is considered a positive indicator of vitality. MIC values were taken as the lowest concentration of the prodigiosin in the wells of those microtiter plates that showed no color change after incubation

##### 2.2.4.4 Time–Kill Curve Analyses

A time–kill assay was performed to determine the time-dependent antibacterial activity of the prodigiosin according to the American Society for Testing and Materials Standards.^16^ Concentrations of prodigiosin were prepared 10 times the MIC value. *S. aureus* was grown to a log phase, and the final inoculum was approximately 10^5^ cfu/mL. After the incubation period, prodigiosin samples were removed from each bottle at 0, 1, 2, 3, 5, and 15 and 1, 3, 6, 12, and 24 h; serially diluted; and plated on tryptic soy agar plates for enumeration of viable colonies by the spread plate count technique. Plates were incubated for 48 h at 37 °C. Colony counts were determined, and the log10 reductions were calculated. All antibacterial tests were repeated three times.

#### 2.2.5 *In Vivo* Epicutaneous Infection Model

##### 2.2.5.1 Animals and Experimental Design

The study was approved by the Ege University, Local Ethical Committee of Animal Experiments (26.01.2022, 2022–010). Ethical guidelines for the investigation of experimental pain in conscious animals were considered in all *in vivo* experiments.^17^ Wistar albino rats (weighing 200–250 g, equivalent to 8–10 weeks of age) were obtained from Ege University Center for Research on Laboratory Animals (Izmir, Turkey). Animals were housed under specific controlled pathogen-free conditions consisting of a 12h light/dark cycle, a temperature of 22 °C, a relative humidity of 40%, and free access to water and food. Before starting the experiment, a week was given to acclimate to the new environment.

##### 2.2.5.2 *S. aureus* Suspension Preparation

*S. aureus* was plated on a TSA plate and grown for 24 h at 36 °C. Bacterial colonies were picked and cultured in TSB overnight at 36 °C. Mid-logarithmic phase bacteria were obtained. The bacterial pellet was washed three times and resuspended in PBS at 5 × 10^8^ cfu/100 μL.

##### 2.2.5.3 Infection Model and Collection of Swap Samples

*S. aureus* epicutaneous infection, which was previously described, was used as an *in vivo* model.^18^ At the start of the experiment, rats were randomly assigned to control and prodigiosin groups (*n* = 18). Rats treated with sterile physiologic serum were the negative control (G1) (*n* = 6). Rats treated with *S. aureus* were used as an infection model (G2) (*n* = 6). The final group was treated with *S. aureus* and prodigiosin as the healing reference (G3) (*n* = 6). Under anesthesia, the dorsal skin of rats was removed, and a 100 μL volume of 5 × 10^8^ cfu *S. aureus* was applied epicutaneously. After the infection had occurred, 100 μL of prodigiosin was used. Swap samples were collected at 0, 1, 6, 24, and 48 h, and the swaps were inoculated onto a Baird-Parker agar. Plates were incubated at 36 ± 1 °C for 48 h. At the end of the incubation period, colonies were counted (30–330 cfu/plate).

The severity of skin inflammation was quantified using a total disease score, which is the sum of the individual grades for erythema, edema, erosion, and scaling, where each was graded as 0 (none), 1 (mild), 2 (moderate), or 3 (severe).^19^

At the end of the experiment, animals were sacrificed, and the routine protocols were performed for evaluation of histological analyses.

#### 2.2.6 Histopathological Examination

##### 2.2.6.1 Tissue Processing and Microscopy

The excised skin samples were subjected to the routine tissue processing procedure. Samples fixed in 4% paraformaldehyde were dehydrated in a series of increasing percentages of alcohol. Tissues cleaned with xylene were embedded in paraffin to obtain paraffin blocks, and the blocks were cut at 5 μm. Sections were dewaxed by soaking them in xylene overnight followed by hydration with a graded alcohol series. All stained sections were cover-slipped after mounting. Imaging was performed on a camera (Olympus DP72, Tokyo, Japan) connected to a microscope (Olympus BX51, Tokyo, Japan) with a histological analysis program (CellSens Software, Olympus). The samples stained with hematoxylin–eosin (H&E), Masson trichrome, and Movat pentachrome dyes were examined under a light microscope at 200× (total) and photographed at 100× (total) magnification.

##### 2.2.6.2 Hematoxylin–Eosin Staining

Histomorphological changes in the cell and tissue structure were investigated with H&E staining that detailed the nucleus in blue/purple color and the remaining tissue in sequential pink color. Tissue sections were deparaffinized in xylene and hydrated through a descending alcohol series from 100%. After staining with hematoxylin, differentiation and rapid bluing steps were applied. Then, the sections dyed with eosin were cleared with fresh xylene, mounted with the mounting medium, and covered with a slip.

##### 2.2.6.3 Masson Trichrome Staining

Dystrophic and fibrotic changes in the tissue were visualized by Masson trichrome stain (04-011802, BioOptica), where cytoplasm and muscle fibers were displayed red, whereas collagen was colored blue. In brief, dewaxed sections were subjected to the following application steps, respectively: rehydrated through descending alcohol series; refixed in Bouin’s solution; colored with Weigert’s iron hematoxylin; dipped in Biebrich scarlet-acid fuchsin; differentiated in phosphomolybdic–phosphotungstic acid; dyed with aniline blue and differentiated in acetic acid; at last, dehydrated through ascending alcohol series; and cleared in xylene. Stained sections were mounted with a mounting medium and cover-slipped.

##### 2.2.6.4 Movat Pentachrome Staining

Tissue sections were stained with the Movat pentachrome stain kit (Modified Russel-Movat, MPS2, Scytek), which enables multifactorial comparison with simultaneous staining of elastin, collagen, muscle, mucin, and fibrin. In brief, an elastic stain solution was prepared from a mixture of hematoxylin, ferric chloride, and Lugol’s iodine solution. Paraffin-free sections were treated with the elastic stain solution for 20 min. Subsequently, it was treated with iron chloride differentiation solution, sodium thiosulfate solution, acetic acid solution, and Alcian blue solution. Differentiation was done with Biebrich scarlet–acid fuchsin, acetic acid, and phosphotungstic acid solutions. Distilled water and/or tap water was used for washing. Finally, the slides were rinsed at alcohol changes and sealed with an occlusion medium.

### 2.3 Statistical Analysis

Statistical analyses were performed by using SPSS for Windows 10.0 and the GraphPad Prism v8.0 statistical analysis program. The results were compared according to the control group using the Student’s *T* test. Values were expressed as mean ± SD. Values of *P* < 0.05 were considered statistically significant.

## 3 Results

### 3.1 Prodigiosin Production, NMR, and TLC Analysis Results

**Figure 1:**
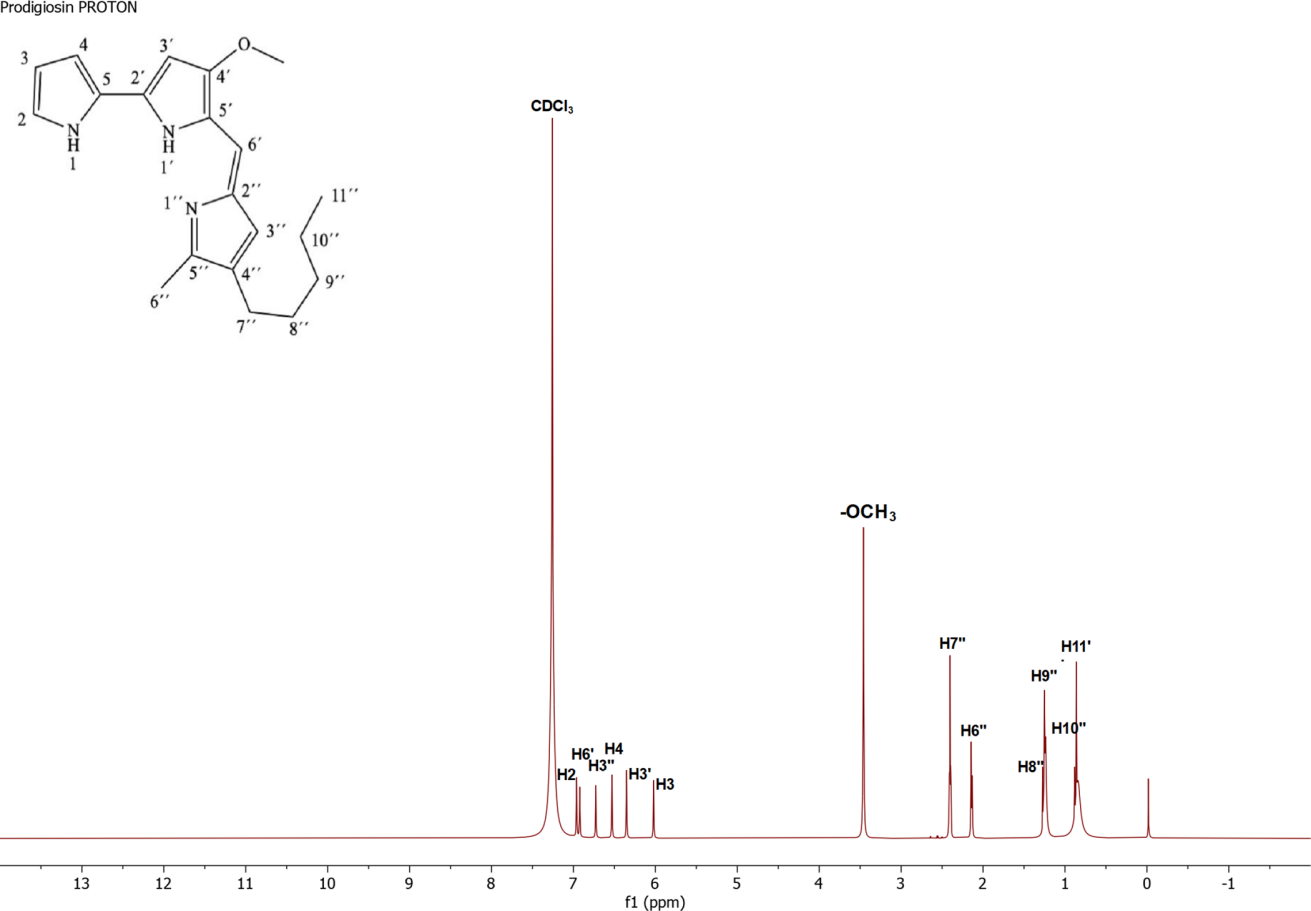
^1^H NMR Spectrum of prodigiosin in CDCl_3_.

Production and identification of secondary compounds can be defined as the first procedure applied to micro-organisms to establish the presence or absence of given analytes. Prodigiosin was produced by *S. marcescens* via 150 mL liquid fermentation for 3 days. After fermentation, the TLC bioautographic assay was performed to determine the prodigiosin. As a result of the TLC analysis, one spot was observed, and the Rf value of this spot (0.9) was in good agreement with the literature (Maurya et al., 2020). The peaks in the ^1^H NMR spectrum are (CDCl_3_), δ (ppm): 0.86 (3H, t, H11″), 1.24 (2H, m, H10″), 1.25 (2H, m, H9″), 1.27 (2H, m, H8″), 2.14 (2H, s, H6″), 2.40 (2H, t, H7″), 3.46 (3H, s, -OCH_3_), 6.02 (1H, m, H3), 6.35 (1H, s, H3′), 6.53 (1H, s, H4), 6.73 (1H, s, H3″), 6.92 (1H, s, H6′), 6.96 (1H, m, H2). These peaks are in good agreement with the literature.^20^ The results of the current study on the characterization of prodigiosin are consistent with a previous work by El-Batal et al.^21^ Thus, it was proved that the pigment was prodigiosin (Figure 1).

### 3.2 *In Vitro* Antibacterial Test Results

**Figure 2:**
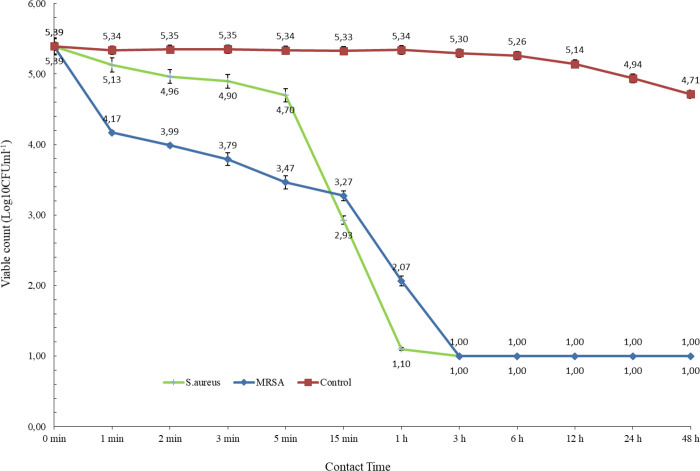
Time–kill kinetics of bacterial suspensions treated with prodigiosin.

Susceptibility testing was performed *in vitro* to confirm the activity of the prodigiosin in a static test situation. The usual limits for counting bacteria on agar plates are between 15 and 300. According to the MIC method, prodigiosin displayed antibacterial activity at 5.5 μg/mL against *S. aureus* and 11 μg/mL against MRSA (Table 1). The agar well diffusion results indicated that prodigiosin showed the most expansive zone at 29.4 mm for *S. aureus* and 25.6 mm for MRSA. In ampicillin as a positive control, zone diameters of 26.7 mm for *S. aureus* and 23.8 mm for MRSA were measured (Table 1, Figure 2).

**Table 1:** Minimum inhibitory concentration of Prodigiosin against *Staphylococcus aureus* strains at μg/mL

	MIC (μg/mL)											
micro-organisms	1500	750	375	187	93	46.5	23.25	11	5.5	2.75	negative control	positive control
*S. aureus*	-	-	-	-	-	-	-	-	-	+	-	+
MRSA	-	-	-	-	-	-	-	-	+	+	-	+

### 3.3 *In Vivo* Epicutaneous Infection Model Result

**Figure 3:**
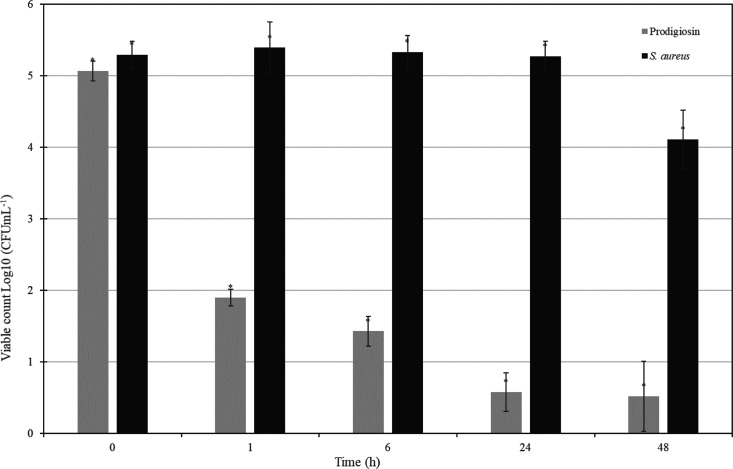
Time-dependent antibacterial activity of prodigiosin against *S. aureus*.

In this model, rats were challenged to develop small purulent lesions with predictable areas of inflammation that mimicked a human skin infection. Rats were infected via epicutaneous challenge with *S. aureus*, and each rat was controlled post-infection to determine lesion development and severity. Swap samples were collected at 0, 1, 6, 24, and 48 h. Results demonstrated that severe skin inflammation caused by *S. aureus* was detected in the infection control group after 2 days post-infection. The surfaces of the infected skins were detected as red and swollen, and total scores were calculated as 3 (severe). The swap samples collected from the *S. aureus* control group were inoculated. After the calculation of colonies, it was determined that there was not any statistical decrease. A decrease in the size and severity of lesions was observed after prodigiosin treatment at 24 h. After 48 h, there was no sign of inflammation, red points, or crusted areas for the prodigiosin group. The severity of skin inflammation was quantified as 0 (none). Swap samples indicated that prodigiosin exposure to *S. aureus*-infected skin caused five logarithmic reductions. There was no erythema, edema, erosion, or scaling in the negative control group, which was treated with sterile physiologic serum (Figure 3).

### 3.4 Histopathological Examination

The control skin sections consisted of a smooth epidermis and underlying well-arranged dermis with primary appendages such as sebaceous glands, apocrine glands, and hair follicles. Dyskeratosis, characterized by nuclear pyknosis and hyper-eosinophilic cytoplasm, and spongiotic changes (spongiosis), distinguished by intercellular space formation due to edema in the epidermal cells and epidermis, were prominent in G2 sections. The epidermis was two to three cells thick in healthy rats; however, distinct epidermal thickening characterized by hyperkeratosis and parakeratosis was notable at the infection site in G2. Basal cell proliferation formed exophytic protrusion (nodule) in the skin; however, this did not result in ulceration on the surface epithelium. Furthermore, the Haarscheibe of the epidermis, which is postulated to be a sensory touch receptor concomitant to the concentration area of Merkel cells, was altered.

**Figure 4:**
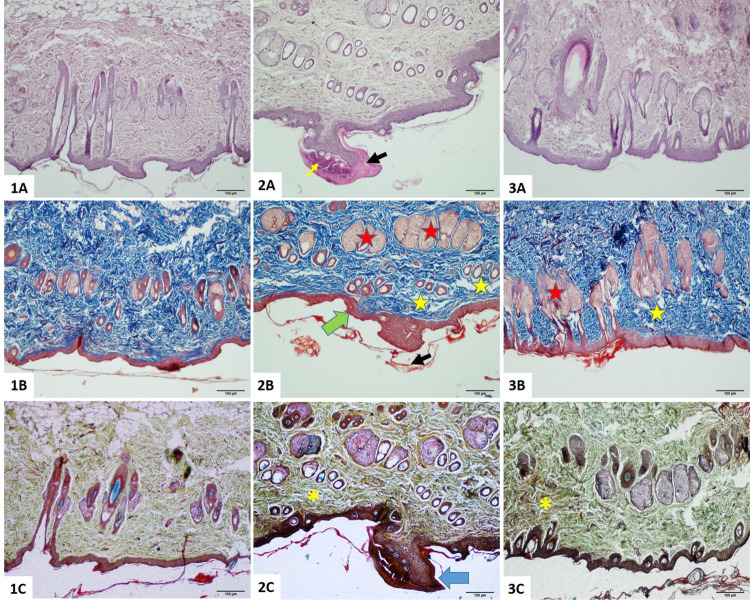
Skin sections of study groups (1–3) were stained with (A) H&E, (B) Masson trichrome, and (C) Movat pentachrome, respectively. (1A–C) Control skin sections consist of a smooth epidermis and underlying well-arranged dermis with primary appendages such as sebaceous glands, apocrine glands, and hair follicles. (2A–C) Epidermal thickening characterized by hyperkeratosis and parakeratosis is accompanied by spongiosis, hyperplastic sebaceous glands, basal cell proliferation with hyperchromatic nuclei, increased inflammatory cell infiltrates with edema, and irregularly dispersed collagen fibers. (3A–C) Improved histopathological findings with hyperplastic sebaceous glands and partial disorganization of connective tissue. Total magnification: 100×. Scale bar: 100 μm. Black arrow: wound site with thickened epidermis. Blue arrow: hyperkeratosis. Green arrow: parakeratosis. Yellow star: spongiotic changes. Red star: hyperplastic sebaceous glands. Yellow arrow: inflammatory cell infiltrate. Yellow asterisk: irregularly dispersed collagen fibers.

Contrary to this, G3 showed more prominent Haarscheibe. Sebaceous gland hyperplasia was accompanied by increased inflammatory responses ranging from prominent neutrophil infiltrates to mixed inflammatory cell infiltrations with edema and extrusion among the wound site in G2; however, hyperplastic sebaceous glands and the expansile lesion were regressed in G3. The altered cell pleomorphism and cellularity, as well as irregularly dispersed collagen fibers, were more notable in the connective tissue of G2 than in G3 (Figure 4).

## 4 Discussion

The skin plays a significant role in coordinating immune responses, contributing to homeostatic maintenance, and disrupting beneficial host–micro-organism interactions. However, bacterial infections cause damage to skin integrity. It is especially known that *S. aureus* epicutaneous exposure drives skin infection, atopic dermatitis, eczema, and inflammation and spreads throughout the blood, triggering a potential life.^22^ Struggle with persistent, localized symptoms of a *S. aureus* infection could be challenging under stress conditions such as tissue injury or chronic inflammation. Although many pharmaceuticals are commonly used in the treatment of bacterial infections, incorrect usage, adverse effects, and drug resistance could be the major reason to search for alternative approaches.^23^

The role of bioactive metabolites of micro-organisms as a source of remedies has been recognized for many years. As a result of the aforementioned side effects, the study of the use of secondary metabolites of microbial origin is gaining worldwide importance.

This study provides a new perspective on treating *S. aureus*-caused infection by prodigiosin isolated from actinomycetes, *S. marcences*. The first step of this study was designed to evaluate the synthesis, isolation, identification, and structural elucidation of prodigiosin. Bioautography refers to a method of testing for micro-organisms commonly used to detect antimicrobial activity. Hence, the isolated prodigiosin from *S. marcescens* was purified by the TLC method successfully. *In vitro* antibacterial methods represented the biological efficacy of prodigiosin. The results of MIC of the potent prodigiosin were observed in 5.5 and 11 μg/mL concentrations against *S. aureus* and MRSA, respectively. The maximum zone of bacterial inhibition for prodigiosin with 29.4 mm for *S. aureus* and 25.6 mm for MRSA was observed. In addition, when the agar diffusion results are compared with 10 μg/mL ampicillin (23.8 mm) as the positive control, it is clearly seen that the inhibition effect of prodigiosin on MRSA is higher. Time–kill assay was performed to determine the bactericidal kinetics of prodigiosin against *S. aureus* and MRSA. The investigation revealed that prodigiosin induced a 3 log10 reduction in the test strains within 1 h, and by the conclusion of a 3 h exposure, it completely inhibited all the inoculated micro-organisms. Prodigiosin has previously been shown to have antibacterial effects against Gram-positive and Gram-negative bacteria.^3,24^ These studies proved that prodigiosin has antistaphylococcal and anti-MRSA activity. In the research conducted by Yip et al., it was observed that a minimal concentration of 10 μg/μL prodigiosin inhibitor was necessary to impede the proliferation of *S. aureus*, *Escherichia coli*, and *E. faecalis*. In contrast, a concentration exceeding 10 μg/μL was found essential in restraining the growth of MRSA.^25^ In the light of current studies, our findings supported the fact that prodigiosin showed the greatest activity for various types of pathogenic bacteria, and it is a promising microbial metabolite with multiple bioactivities.^4,7,26^

The literature shows that the best analysis to understand *in vitro* tests is predictive of *in vivo* animal studies.^27^ Animal infection models are an integral part of host–pathogen research and are used to approximate the complex environment of the human body. *In vivo*, the epicutaneous infection model represents the suppression effects of antimicrobial compounds. This study aimed to determine whether the tested prodigiosin had a potent antibacterial effect against *S. aureus*. It was conducted on rats and supported by swap analyses and histopathological examinations. The results suggest that prodigiosin exposure inhibits the *S. aureus*-caused skin infection after 48 h. When comparing the healthy rat’s skin microflora with the prodigiosin group, it was shown that both swap samples had negative colony results. Disruption of the integrity of the skin by *S. aureus*, which is one of the most common harmful bacteria of skin and soft tissue infection, was examined. When comparing the control group, it was shown that prodigiosin inhibits the bacterial infection successfully according to selected times. The histopathological samples demonstrated that microbial colonies decreased significantly, and an anti-*S. aureus* effect was exhibited for prodigiosin. Prodigiosin characterization from different strains and evaluation of the biological activities such as anticancer, neuroprotective, wound healing, anti-algicidal properties has been reported.^6,8^ This is the first study that focused on the prodigiosin *S. aureus* inhibition effect on an epicutaneous infection model.

## 5 Conclusions

This study indicates that prodigiosin is an antibacterial compound, thereby showing great potential for epicutaneous infection caused by *Staphylococcus*. The development of a thin-layer chromatography bioautographic assay and NMR analysis for prodigiosin proved that the purified active compound demonstrated antibacterial efficacy against *Staphylococcus. In vivo,* animal studies verified that prodigiosin has high potential bioactivity and is efficient for bacterial epicutaneous infections. Besides the many health benefits of prodigiosin, it was predicted that it could be used as an active drug ingredient or as a combination of therapeutic interventions in infection care. Thus, our findings hold a great promise for the use of prodigiosin as supportive therapy that deserves to be explored further.

## Notes

The study was approved by the Ege University, Local Ethical Committee of Animal Experiments (26.01.2022, 2022–010).
